# Morphodynamic evolution following sediment release from the world’s largest dam removal

**DOI:** 10.1038/s41598-018-30817-8

**Published:** 2018-09-05

**Authors:** Andrew C. Ritchie, Jonathan A. Warrick, Amy E. East, Christopher S. Magirl, Andrew W. Stevens, Jennifer A. Bountry, Timothy J. Randle, Christopher A. Curran, Robert C. Hilldale, Jeffrey J. Duda, Guy R. Gelfenbaum, Ian M. Miller, George R. Pess, Melissa M. Foley, Randall McCoy, Andrea S. Ogston

**Affiliations:** 1 Pacific Coastal and Marine Science Center, United States Geological Survey, Santa Cruz, CA USA; 2 Arizona Water Science Center, United States Geological Survey, Tucson, AZ USA; 3 Sedimentation and River Hydraulics Group, Technical Service Center, United States Bureau of Reclamation, Denver, CO USA; 4 Washington Water Science Center, United States Geological Survey, Tacoma, WA USA; 5 Western Fisheries Research Center, United States Geological Survey, Seattle, WA USA; 6 Washington Sea Grant, Olympic Peninsula Field Office, Port Angeles, WA USA; 7 Northwest Fisheries Science Center, National Marine Fisheries Service, National Oceanic and Atmospheric Administration, Department of Commerce, Seattle, WA USA; 8 Natural Resources Department, Lower Elwha Klallam Tribe, Port Angeles, WA USA; 9 School of Oceanography, University of Washington, Seattle, WA USA

## Abstract

Sediment pulses can cause widespread, complex changes to rivers and coastal regions. Quantifying landscape response to sediment-supply changes is a long-standing problem in geomorphology, but the unanticipated nature of most sediment pulses rarely allows for detailed measurement of associated landscape processes and evolution. The intentional removal of two large dams on the Elwha River (Washington, USA) exposed ~30 Mt of impounded sediment to fluvial erosion, presenting a unique opportunity to quantify source-to-sink river and coastal responses to a massive sediment-source perturbation. Here we evaluate geomorphic evolution during and after the sediment pulse, presenting a 5-year sediment budget and morphodynamic analysis of the Elwha River and its delta. Approximately 65% of the sediment was eroded, of which only ~10% was deposited in the fluvial system. This restored fluvial supply of sand, gravel, and wood substantially changed the channel morphology. The remaining ~90% of the released sediment was transported to the coast, causing ~60 ha of delta growth. Although metrics of geomorphic change did not follow simple time-coherent paths, many signals peaked 1–2 years after the start of dam removal, indicating combined impulse and step-change disturbance responses.

## Introduction

**Figure 1:**
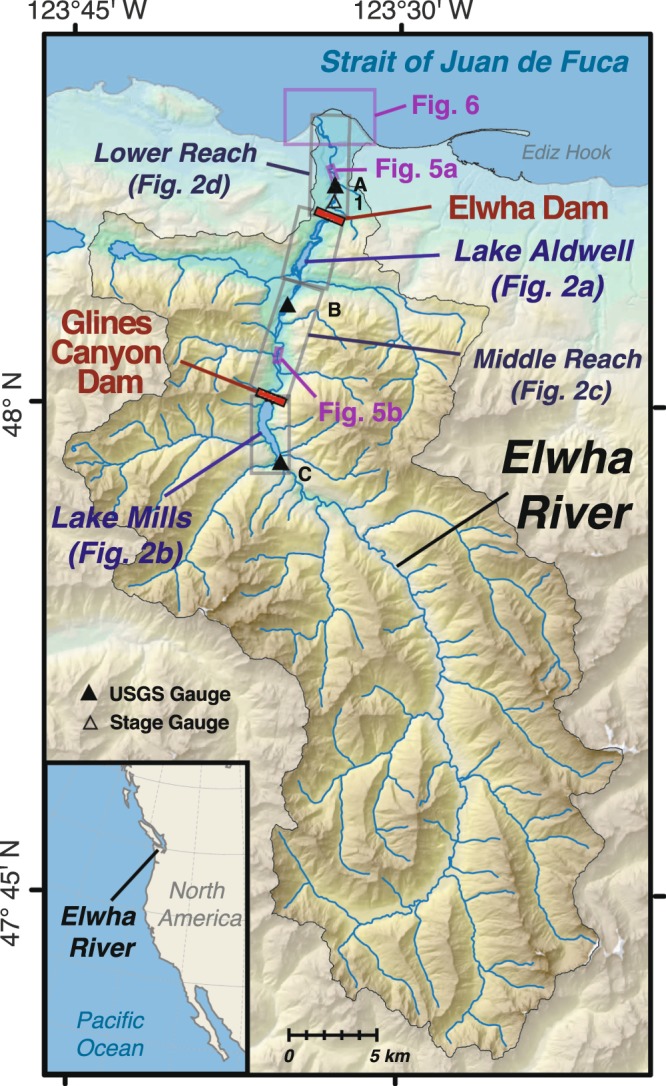
Map of the Elwha River watershed (location in inset), showing the dam and reservoir locations, river gauging stations (triangles), and inset locations of figures. Stations include USGS stream gauges 12046260 (A), 12045500 (B) and 12044900 (C), and the stage gauge at Rkm 5.5 (1). Base map created with ArcMap version 10 (http://desktop.arcgis.com/en/arcmap/) using data from the USGS National Map (Map services and data available from U.S. Geological Survey, National Geospatial Program).

The Elwha River drains 833 km^2^ of steep alpine and forested terrain within Olympic National Park, Washington, a UNESCO World Heritage Site^1^ (Fig. 1). The world’s largest dam removal in terms of dam height and reservoir-sediment volume^2^ occurred on the Elwha River through the simultaneously initiated, phased removal of two dams between 2011 and 2014—Elwha Dam (32 m tall; 7.9 km from the river mouth), and Glines Canyon Dam (64 m high; 21.6 km from the mouth; Fig. 1). This exposed ~30 Mt of sediment trapped in the reservoirs during their 84- and 98-yr lifespans^3^. Natural fluvial erosion of the reservoir sediment during and after the dam removals renewed sediment and wood fluxes to the downstream river and coast, where effects of reduced sediment supply had been evident while the river was dammed^4,5^. The removal of both dams on the Elwha River released a sediment volume 5-fold greater than the next-largest dam removal^2,6^, creating a fluvial sediment pulse comparable in sediment-source area, sediment yield, and watershed area to that in rivers affected by the 1980 Mount St. Helens volcanic eruption^7^.

Fluvial sediment pulses can influence riverine characteristics markedly, including water quality, channel and floodplain morphology, habitats, ecosystems, groundwater elevations and transmissivity, and flooding hazards^2,8–13^. Downstream morphodynamic responses to dam removals can resemble effects ranging from those of a small landslide to a modest volcanic eruption, depending on the scale of the disturbance and the area of interest^14–16^. Expectations of landscape responses to such perturbations are based on theory, laboratory and flume experiments, numerical models, and some field studies, most of which are limited in temporal and spatial scale^14,16–19^. Physical responses to dam removal depend on numerous factors, including the composition and quantity of sediment and organic material released, and the hydrology and geomorphology of the watershed and its coastal delta. These responses typically include (i) reservoir sediment erosion by channel incision and lateral migration due to lowered base level^20^, (ii) increased sediment supply downstream of the source region, including an abrupt increase in bed-material transport that fills pools and locally increases bed elevation and river slope between riffles^18,21–23^, (iii) increased channel width, braiding, and rates of channel migration—effects that extend into the floodplain^20,24,25^—, and (iv) a non-linear approach toward geomorphic equilibrium that may or may not resemble the pre-dammed state^17,26,27^.

Little information exists regarding source-to-sink sedimentary and geomorphic effects of large dam removals, especially over multi-year scales before and after removal^28^. We provide novel insights by quantifying morphodynamic effects and developing a 5-year, source-to-sink sediment budget for the Elwha River. Our study measured the magnitude, extent, and duration of landscape disturbance induced by an intentional sediment pulse comprehensively, at unprecedented spatial and temporal resolutions.

## Sediment Supply from Dam Removal

**Figure 2:**
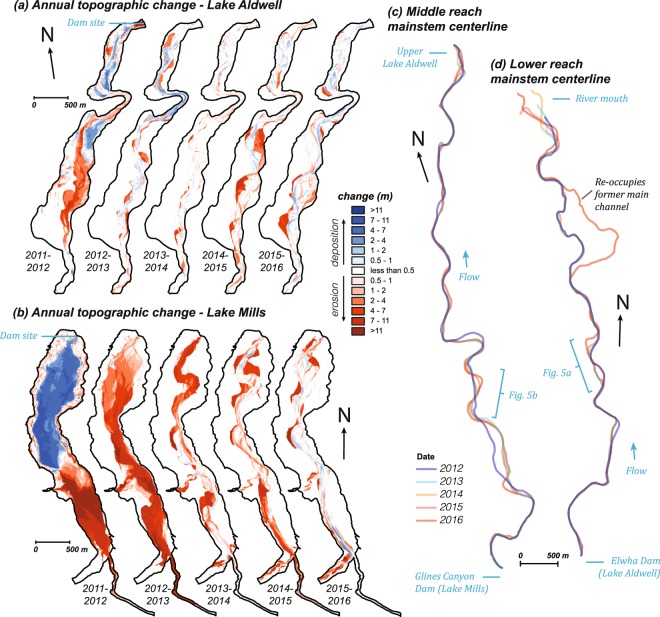
(**a**,**b**) Maps of topographic change between annual summer low flows in the two reservoirs of the Elwha River during and following dam removal: (**a**) Lake Aldwell and (**b**) Lake Mills. (**c**,**d**) Maps of the Elwha River thalweg during annual summer low flow for (**c**) the middle reach, which lies between the two dam sites, and (**d**) the lower reach, which lies between the Elwha Dam site and the coast. Figure created with ArcMap version 10.5 (http://desktop.arcgis.com/en/arcmap/) using structure-from-motion products created with Agisoft PhotoScan 1.1.6 through 1.2.6 (http://www.agisoft.com).

The greatest topographic changes resulting from the Elwha River dam removals occurred in the two former reservoirs, which transitioned from lacustrine depocenters to fluvial source, transport, and storage reaches, and through which the river eroded and redistributed sediment and exported it downstream (Fig. 2a,b). Substantial sediment redistribution within the reservoirs occurred in the first year of dam removal, as nearly all eroded sediment from the upstream portion of the Lake Mills delta (formerly impounded by Glines Canyon Dam) was redeposited within the shrinking reservoir (Fig. 2b). Similarly, ~42% of eroded sediment from the Lake Aldwell delta (impounded by Elwha Dam) was redeposited on its former lakebed (Fig. 2a), with the remainder exported downstream.

**Figure 3:**
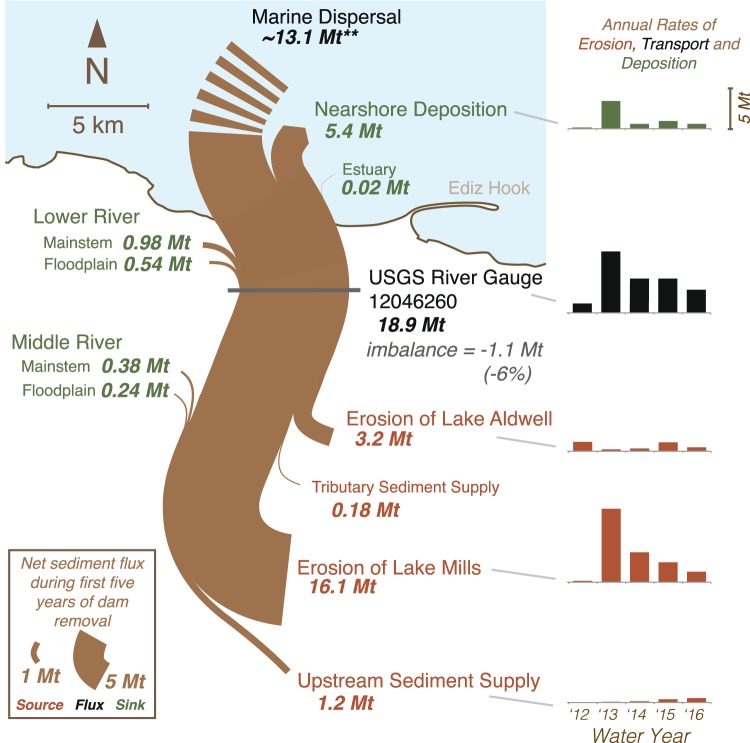
Sediment budget for the Elwha River and coast during the first five years of the dam removal (water year [WY] 2012 to 2016). Line thickness is scaled to the five-year flux values. Annual time series of fluxes are shown on the right-hand panel (scale at top-right). Uncertainty values for each element are provided in Supplementary Table 1. Sediment lost to the Strait of Juan de Fuca by marine dispersal (**) was calculated by mass balance and has 2-σ uncertainty of 3.7 Mt.

**Figure 4:**
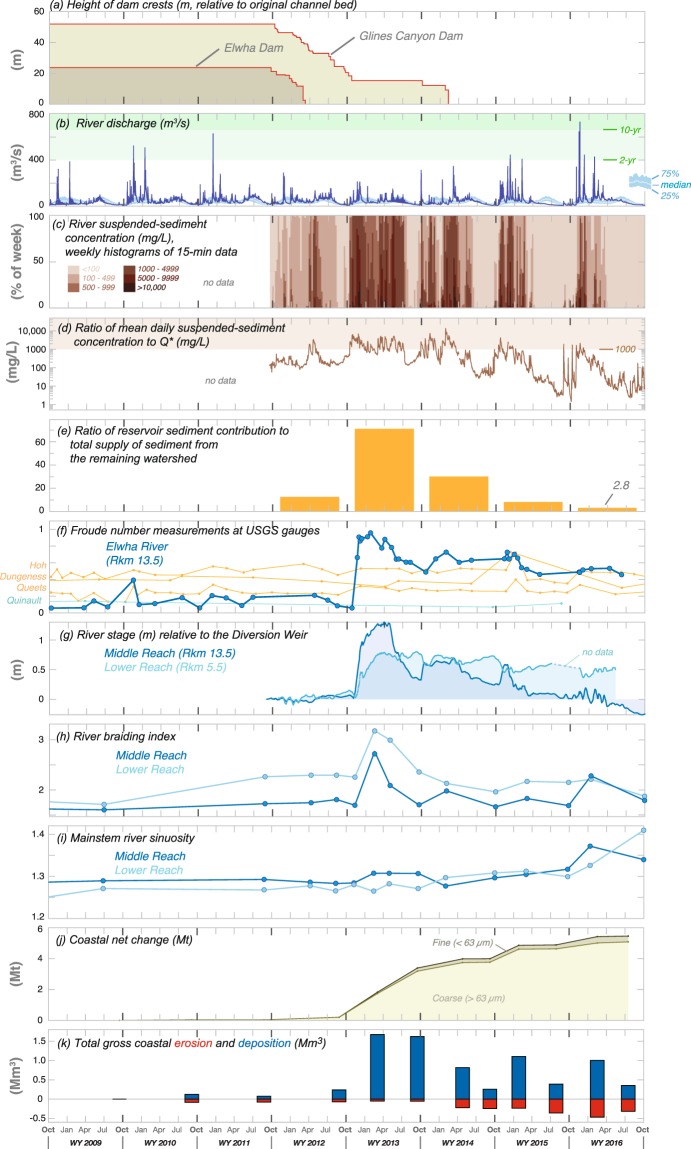
Eight-year time series of metrics used to define the magnitudes and time scales of river and coastal response to dam removal on the Elwha River (see Methods for descriptions of metrics).

The reservoir-derived sediment pulse peaked during the second year of dam removal, when the reservoirs supplied ~70 times more sediment than the watershed’s estimated natural sediment load (Figs 3 and 4e). During the second year of dam removal, Lake Mills became the dominant source of sediment to the river and coast, exporting 8.8 ± 1.8 Mt of sediment (annual rates in Fig. 3; 2-σ uncertainty provided throughout as tabulated in Supplementary Table 1) as the river incised >10 m and laterally migrated 100 s of meters within the former reservoir, eroding terraces as it reworked and exported sediment impounded during the dammed era (Fig. 2b). The abundant sediment supply from Lake Mills increased deposition in and slowed the rate of net erosion from Lake Aldwell, which exported only 0.5 ± 0.1 Mt of sediment during the combined second and third years (Fig. 2a, annual rates in Fig. 3). At the end of the fifth year (fall 2016), 19.3 ± 3.8 Mt of sediment (~65% of the initial sediment mass) had been exported from the two reservoirs, increasing the 5-year sediment yield by an order of magnitude above the estimated natural yield (Figs 3 and 4e; uncertainty estimates in Supplementary Table 1).

During these 5 years (water years 2012–2016), a combined ~20.5 ± 3.2 Mt of sediment was supplied by the reservoirs and the upstream watershed. We estimate that of this sediment, ~10% (2.1 ± 0.4 Mt) was deposited in the river channel and floodplain, while ~26% (5.4 ± 1.6 Mt) was deposited in and around the coastal delta (Fig. 3). The remaining ~64% (13.0 ± 3.2 Mt) was transported offshore beyond the limits of the coastal bathymetric surveys (beyond the 15 m isobath).

Thus, the Elwha River efficiently eroded and transported sediment from its reservoirs, through the fluvial system, and to the coast. The efficiency with which the Elwha River transported sediment from source to sink is remarkable given that flows were generally below the historical mean when most sediment was eroded (Fig. 4b,e). During the first three years after dam removal began, annual peak flows were well below the 2-year flood magnitude (Fig. 4b), and snowmelt flows were near the historical mean^1^ (Fig. 4b). Nevertheless, sediment erosion surpassed predictions developed from numerical and physical models, which had indicated that less than half of the stored reservoir sediment would erode^24,29,30^. This finding illustrates the continuing challenge of predicting sediment transport and large-scale geomorphic processes, and suggests that scaling effects and model simplifications^17,31,32^ under-represented the capacity of the Elwha River to laterally erode sediment from reservoir deposits. Several other notable dam removals have eroded similar percentages of their reservoir deposits in just a few years^11^.

## Downstream Effects of Renewed Sediment Supply

As the river exported this massive sediment pulse from the reservoirs, fluvial suspended-sediment concentrations (SSC) measured below both reservoirs (at USGS gauge 12046260; labeled ‘A’ in Fig. 1) were continuously 100 s to 1000 s of mg/L for weeks to months, especially during the second year of dam removal (WY 2013; Fig. 4c). These concentrations exceeded those measured before dam removal by up to several orders of magnitude, and were greatest during winter high flows and snowmelt peaks. However, the SSC values decreased with time following complete removal of both dams and especially during summer low flow, as shown by values normalized by the ratio of measured river discharge to mean annual flow (SSC/Q*) (Fig. 4d). Bed-material transport was similarly greatest during high flows after bed sediment began to pass both dam sites (early in WY 2013; see Supplementary Information).

**Figure 5:**
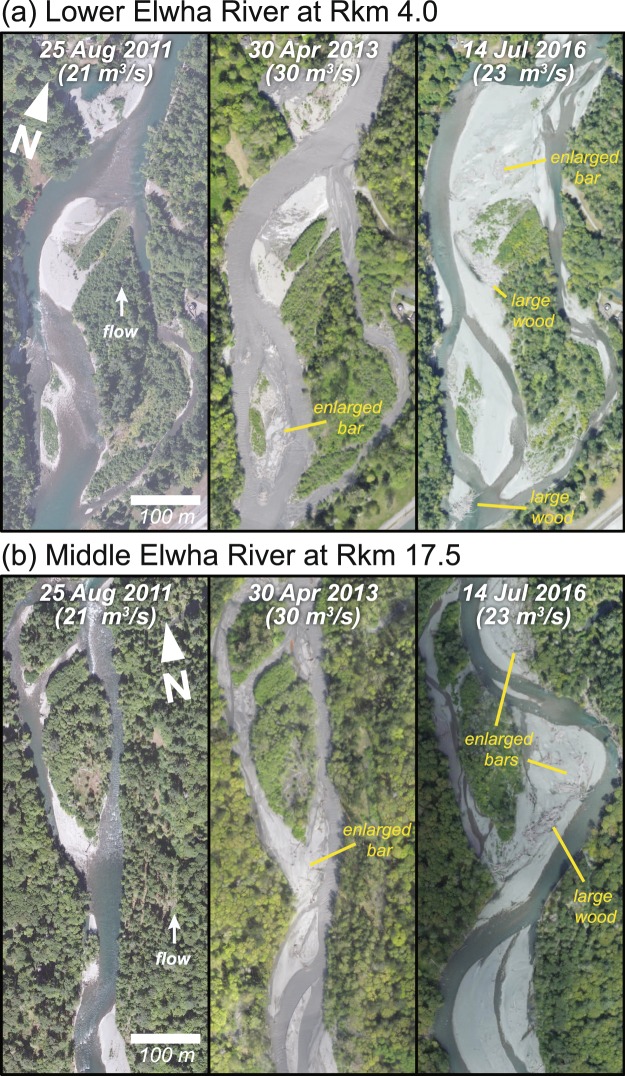
Aerial orthophotos from the (**a**) lower and (**b**) middle reaches of the Elwha River, showing geomorphic effects of sediment and wood release from dam removal. Photos are provided for before dam removal (25 Aug., 2011; left panel), during the peak sediment release in spring 2013 (30 Apr., 2013; middle panel) and approximately 5 years after dam removal was initiated (14 Jul., 2016; right panel). Figure created with ArcMap version 10.5 (http://desktop.arcgis.com/en/arcmap/) using structure-from-motion products created with Agisoft PhotoScan 1.1.6 through 1.2.6 (http://www.agisoft.com). Left panels are National Aerial Imagery Program data (U. S. Department of Agriculture, Farm Service Agency).

The new sediment supply—along with a renewed supply of large wood^22,33^—fundamentally changed the geomorphic form and flow conditions of the river. Riverbed pools filled with sediment, smoothing the longitudinal profile^22^ as the river channel aggraded substantially (Fig. 4g) and became more braided^22,33^ (Fig. 4h). These changes coincided with pool filling, sediment-bar growth, and large-wood deposition throughout the river below the dam sites (Fig. 5). Froude number (the ratio of inertial and gravitational forces within a flow) and water-surface elevation (stage) independently demonstrated channel response to increased sediment supply at discrete points in the river, showing coincident increases in the first half of WY 2013 (Fig. 4f,g). The changes in Froude number at the Elwha River gauging station were unlike any measured contemporaneously for nearby rivers of the Olympic Mountains, which remained relatively stable (Fig. 4f). Aerial surveys, water-level monitoring, and topographic surveys confirmed that discrete measurements of changes in stage were attributable to 1.0–1.5 m of widespread riverbed aggradation^22^, rather than a localized response. Moreover, these extensive changes in river form and process occurred with flows substantially less than the 2-year flood discharge (Fig. 4b).

The sedimentary and geomorphic signal of dam removal began to wane during the second year of dam removal (WY 2013). For example, river stage began to decrease as the river incised through the newly deposited sediment (Fig. 4g). The degree of channel braiding decreased rapidly as the sediment pulse waned (Fig. 4h), first in the middle reach (measured from below Glines Canyon Dam to above Lake Aldwell; Fig. 1), then in the lower reach (measured from below Elwha Dam to just above the coastal delta; Fig. 1). Mainstem sinuosity in both reaches (Fig. 4i) showed minimal response during the first two years following dam removal, but increased during the last three years, in concert with higher peak flows (Fig. 4b). As such, it was largely decoupled from other metrics. Sinuosity increase in the lower reach in WY 2015 and 2016 was partially attributable to flow being directed by new engineered log jams down the longer of two major anabranches between 2015 and 2016, whereas in the middle reach increased sinuosity was driven by lateral channel migration, especially in the unconfined floodplain (Fig. 2c,d).

## Expansion of the Elwha River Delta

**Figure 6:**
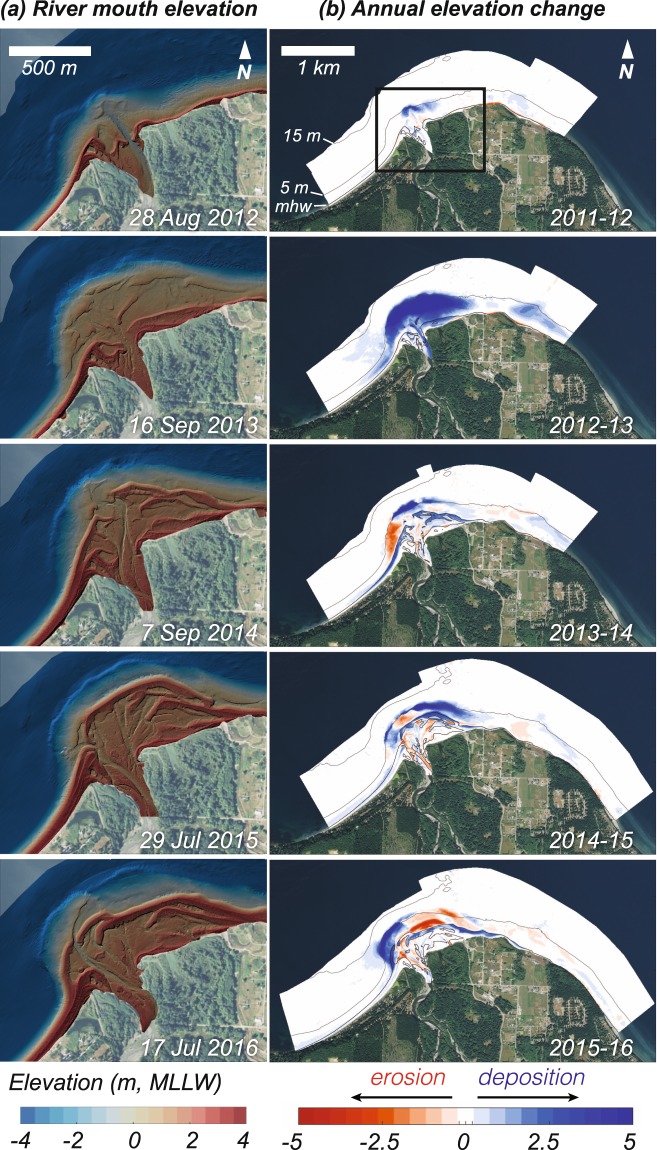
Topographic change of the Elwha River delta during and after dam removal. (**a**) Shaded-relief elevation maps of the river mouth from annual topographic and bathymetric surveys during the summer season. (**b**) Maps of annual change in elevation for the broader Elwha River delta. The three thin black lines on each map show contours of the −15 m, −5 m, and mean high water (mhw) elevations. The box in the upper panel shows the extent of the inset shown in (**a**). Map created with MATLAB Version: 9.2.0.556344 (https://www.mathworks.com). Base map data from the USGS National Map (Map services and data available from U.S. Geological Survey, National Geospatial Program).

The coastal delta area grew by ~60 ha during the first five years of monitoring, demonstrating a fundamental regime shift from coastal erosion to deposition^34,35^ (Figs 4j,k and 6a,b). The 5.4 ± 1.6 Mt of sediment deposited in the coastal delta consisted largely of coarse sediment (sand and gravel), whereas fine sediment represented only ~6% of the total deposited mass (Fig. 4j). This finding is consistent with studies indicating that waves and tidal currents interact with the broader submarine Elwha River delta to produce shear stresses that resuspend and disperse sand and finer sediment seaward and alongshore beyond the delta landform^35–37^.

Sediment deposited not only in the intertidal and subaerial regions of the delta, including in river-mouth bars (Fig. 6a), but also offshore of the river mouth in water as deep as 12 m and in a secondary depocenter ~2 km east of the river mouth (Fig. 6b). Thus, the magnitude and variability of coastal geomorphic change increased dramatically compared to pre-dam-removal conditions, as demonstrated by changes in the gross erosion and deposition rates (Fig. 4k). This increased coastal dynamicity was related largely to reworking of new sediment across and along shore (Fig. 6b). Although coastal response was overwhelmingly depositional (peaking in 2013), local, seasonal erosion occurred nevertheless (Figs 4k and 6b). Coastal erosion, driven by alongshore and across-shore transport, was most pronounced in the summer dry season during low river flow, as sediment delivered to the coast during winter and spring flows was redistributed by waves and currents (Fig. 4k).

## Disturbance Response and Return to “Equilibrium”

The geomorphic effects of a rapid sediment-supply increase are sometimes conceptualized as an impulse response, or a temporary perturbation of an equilibrium followed by non-linear return to the pre-disturbance condition^7,19,38^. Channel perturbations from dam removal result from a pulse input of sediment (and in some systems wood), leading to changes in riverbed slope, elevation, width, sinuosity, and braiding—metrics associated with sediment transport and storage—and may be followed by relaxation of those signals as the sediment pulse wanes and quasi-equilibrium returns (allowing for the river to evolve continually within a natural range of variability). However, an important additional consideration in sediment pulses caused by large dam removals is that the initial disturbance (impulse response) is followed by, or superimposed upon, long-term restoration of upstream sediment and wood supply—analogous to a step response^38^ (a response to an enduring shift in inputs), rather than a simple transient impulse.

In the case of dam emplacement, a step response occurs, reflecting indefinite reduction or cessation of the sediment and wood supply (which can cause downstream incision, bed armoring, channel simplification, and coastal shoreline retreat^39,40^)—effects later essentially reversed by an opposite step response when a reservoir becomes filled, or upon dam removal and return to a more dynamic system^17^. However, in the case of dam removal, the release of accumulated sediment and wood reflects an impulse—with a scale commensurate to the volume of stored material—that overlies (and obscures) the step response.

The response of the Elwha River and its coast to dam-removal disturbance is best understood as an impulse response superimposed upon a step change. Although it is too early to identify a new, decadal-scale geomorphic equilibrium state of the Elwha River, owing largely to the continued influence of former reservoir sediment on the fluvial and coastal sediment budget (Fig. 4e), many geomorphic metrics revealed temporal trends toward equilibrium conditions—some similar to the dammed state and others different—over the first 5 years. Sediment flux waned after an initial 1–2 year pulse, and turbidity, river stage, and channel braiding showed coincident decay of the dam-removal signal (Figs 3 and 4). In contrast, sinuosity increased after the sediment pulse passed. Even after 5 years some post-removal geomorphic metrics remained altered relative to pre-removal conditions—notably Froude number, coastal-delta volume and morphodynamics, and the continual elevation of river stage ~0.5 m above the dammed condition in much of the lower reach (Fig. 4). Taken together, these metrics indicate both a large-magnitude impulse disturbance from rapid release of nearly a century of stored sediment and a lower-order step change from dammed to undammed conditions, reflecting restored sediment and wood flux derived from the upper watershed. Notably, we find that temporal and spatial response signals of the Elwha River dam removals extend longer and farther downstream than those documented in other large dam-removal studies^3,16,18,27^. No other dam-removal research has spanned such an interval or shown such fundamental morphologic shifts—extending up to 5 years and 20 km away from the sediment source.

Comparing the geomorphic effects and longevity of the Elwha River sediment pulse with the effects of fluvial sediment pulses caused by volcanic eruptions and landslides indicates that river resilience to sediment-pulse disturbances varies with sediment grain size (erodibility and cohesion)^41–43^, physiography of the river valley and sediment source area^7,15,43^, channel gradient^7,42^, and hydrology^41,45,46^. Based on our data and literature examples, we infer that rivers in 10^2^–10^3 ^km^2^ watersheds can export sediment pulses of similar or larger magnitude than that of the Elwha River dam removals (~10^7^ t) with similar efficiency (moving >10 km in <5 yr) if the sediment is noncohesive, if flows have sufficient transport power (aided in some cases by hydraulic smoothing during large sediment loads^7^), and where the channel gradient is ~0.003 or steeper^15,41,42,44,45^; the Elwha River gradient below the dam sites is 0.004–0.008. A sediment pulse may cause minimal downstream geomorphic impact and evacuate more rapidly («1 year) even along a channel with 0.003 slope if the sediment pulse contains dominantly silt and clay^43^. Dam removal effectively creates a point-source sediment pulse (from the former reservoir) of fluvial sediment in the river channel, a simpler situation than the non-point-source introduction of sediment following volcanic eruptions or human land use, from which sediment supply often blankets hillslopes over a large area with spatially variable connectivity to the river network^7,47^. A dam-removal sediment pulse is therefore likely to move through a fluvial system more rapidly than a volcanic sediment pulse of similar magnitude.

Other important factors influencing the post-dam-removal evolution of the river and coastline include vegetation changes in the riparian floodplain and coastal delta. The newly altered sediment and wood regime will also continuously interact with aquatic and terrestrial biota. Interplay between river dynamics and riparian and coastal vegetation are well discussed in the literature^48–52^. However, interactions between vegetation and geomorphology typically occur on decadal time scales^49^, and so have not been fully assessed yet in the Elwha system. Similarly, aquatic fauna can be expected to influence Elwha River geomorphology^53,54^, but this must be assessed in longer-term investigations, given that biotic communities respond and reach dynamic equilibrium on longer time scales than the geomorphic changes presented here. However, an increase in pioneering vegetation is already evident at the coastal delta^55^, and changes in pioneer vegetation and wood are apparent in the river (Fig. 5). Shifts have occurred in macroinvertebrate assemblages and subtidal algal communities at the river mouth^55,56^, and new marine-derived nutrients have been documented in the freshwater food web of the Elwha River since dam removal^57^. We anticipate that the river system will continue to respond over decadal scales to renewed downstream fluxes of wood and sediment, and to renewed upstream flux of marine derived nutrients through salmonid migration.

As dam removal becomes increasingly common owing to economic, safety, and environmental factors^28^, the scale and volume of such projects may increase. Results from the largest dam removal to date, on the Elwha River, indicate that the geomorphic effects of a massive sediment release from dam removal can be substantial, but are not necessarily long-lived. However, the complex geomorphic response—strongly dependent on source sediment composition and distribution, channel gradient, hydrology, and physiographic setting—should not be assumed to occur in a simple, coherent manner nor necessarily to diminish rapidly. Dam removal on the Elwha River reveals that fluvial and coastal morphodynamic responses may follow related but nonaligned pathways. We conclude that geomorphic effects of a large-scale dam removal show similarities to both pulsed sediment-supply events (Fig. 4c–e) and a step change in supply (Fig. 4f,k), but do not follow simple time-coherent paths, complicating analysis of the impact duration and signal decay. Although some of these geomorphic impulses decayed within months to years, it remains to be seen how long the detectable impulses in sediment supply will last in the river and coastal systems and what the near-steady-state fluvial and coastal morphology will be following this historic dam removal.

## Methods

The calculation of a 5-yr sediment budget for the Elwha River closely followed published techniques of the 2-yr sediment budget previously presented^3,21,22,33,35^. Here we summarize these techniques, highlighting differences or additional data included in the present results.

The sediment budget was intended to produce annual values of sediment mass flux and/or change (i.e., tonnes/yr) at a series of nodes within the watershed during the dam removals. Annual values focused on hydrologic years (water years, WY, defined from October 1 to September 30) using surveys and sampling that fit these temporal bounds as closely as possible. For the purposes of this paper, “Year 1” referred to the 2012 water year (1 Oct 2011–30 Sept 2012), and so on. All sediment-budget measurements are presented with uncertainty estimated at the 2-σ level^33^. Sediment-budget elements that were derived by summation, such as the marine dispersal, were calculated using the root of the summed squared errors of all inputs, which assumes independent errors among the elements.

Sediment mass changes in the two reservoirs were calculated by integrating several data sets: pre-dam topographic sketches and surveys from 1913 and 1921; pre-dam-removal reservoir topographic and bathymetric surveys and sediment analyses from 1989 and 2010; six aerial lidar surveys of reservoir topography in April 2009, October 2012, November 2014, February 2015, September 2015, and March 2016; structure-from-motion (SfM) photogrammetry of reservoir topography from 5 aerial photographic surveys (at low river flow) in September 2012–2016, and field surveys of landforms and ground control points within the reservoirs and along the river using real-time kinematic global positioning system (RTK-GPS) equipment^3^. The time-dependent volumes of sediment redistribution within the reservoirs, and export from the reservoirs, were calculated from three-dimensional reservoir stratigraphy models that included SfM- and lidar-derived digital elevation models (DEMs) generated at 1-m spatial resolution and two groups of sediment type (sand and gravel, and silt and clay). The model was initialized with data from the 1989 and 2010 surveys and updated using calculations of grain-size-dependent trap efficiency^3^. Conversion of sediment volumes to mass were conducted by applying bulk densities that varied by grain-size^3,33^.

Fluxes of sediment in the river were measured at two gauges (USGS 12044900 – Elwha River above Lake Mills, and USGS 12046260 – Elwha River at Diversion Weir), from which records were supplemented with 15-minute discharge values computed from stage measurements at the long-standing USGS 12045500 Elwha River at McDonald Bridge gauge^21^. For the Diversion Weir gauge, downstream of both reservoirs, near-continuous records of suspended-sediment concentration (SSC) and discharge were calculated using a combination of standard flow-weighted suspended-sediment samples, automated point-sampler daily composite samples, and three sediment-surrogate instruments—two optical turbidimeters and one acoustic Doppler velocity meter (ADVM)—using established techniques^21,58–60^.

The mean daily SSC and river discharge values were used to derive daily-normalized SSC values and loads. Normalization was performed using the methods of Magirl *et al*.^21^, whereby SSC was divided by Q*, the ratio between mean daily discharge and mean annual discharge.

Bedload sediment transport was calculated using a combination of steel bedload-impact plates and physical bedload sampling during November, 2012; March, May, and June 2013; and April 2014 at the Diversion Weir gauge^21,61^. These techniques estimated the proportion of bedload in the >16 mm and 2–16 mm size classes, but did not capture bedload transport of particles smaller than 2 mm, which were estimated from a combination of regional and comparative studies in high-sediment load rivers—including other dam removal studies—using constant ratios of measured total bedload to total sediment discharge of 15% for water year 2012 and 25% for later years. Early in water year 2016, the river avulsed a new channel that partially bypassed the bedload-impact plates and prevented complete bedload measurement. As a result, monthly bedload values for WY 2016 (year 5) were estimated using a discharge rating curve derived from WY 2015 daily measured discharge and bedload >16 mm. Daily values were averaged by month to obtain a rating curve, BL16 = 2.325 * 10^–7^ * Q^5.644^, where BL16 = bedload >16 mm in tonnes/month, and Q = mean monthly discharge at the diversion weir in m^3^/s. Bedload fractions for 2–16 mm and >2 mm size classes, and discharge values at the diversion weir were estimated using the techniques of Magirl *et al*.^21^, as per previous years. Total sediment load at the gauge above Lake Mills was estimated using discharge-related empirical relationships derived from sediment measurements from water years 1994–1998 and 2006–2007^21,62,63^.

Sediment supply from additional watershed areas were estimated using scaled sediment-yield computations on an annual basis. Two areas were included in these estimates: (i) five small, ungauged watersheds draining into Lake Mills (Cat, Boulder, Hurricane, Wolf, and Stukey Creeks), and (ii) two ungauged tributaries of the Elwha River downstream of the Glines Canyon Dam site (Little River and Indian Creek below Lake Sutherland). Tributary inputs into the Lake Mills reach were assessed using the techniques of Magirl *et al*.^21^ and Curran *et al*.^63^, which compared gauged sediment-flux estimates from the aforementioned periods with measurements of reservoir sedimentation from 1926 to 2010. These calculations indicate that the total flux into Mills was 121.5% of that measured at USGS gauge 12044900, a value that implies that sediment yields (t/km^2^) are nearly constant, since the total watershed area draining to Lake Mills is 119% of that gauged by the USGS station. Thus, this additional sediment contribution was added on an annual basis and presented in the total ‘Upstream Sediment Supply’ values in Fig. 3.

For tributaries between Lake Mills and the river mouth, which total 175 km^2^ (21%) of the watershed area, we assumed a similar constant sediment yield, but corrected the sediment yields above Lake Mills for precipitation and landscape gradient using a mean precipitation-weighted stream power index (SPI)^64^, derived using PRISM 30-year precipitation normals^65^ and 1/3 arc-second topography data from the National Elevation Dataset 3D Elevation Program^66^. Because of the lower landscape gradients and precipitation in the tributaries, these calculations indicate that the annual contributions from these tributaries were 15.6% of those above Lake Mills on the basis of specific sediment yield, which is consistent with estimates of denudation in the Olympic Mountains^67,68^. Thus, to estimate tributary sediment supplies on an annual basis, we multiplied the total sediment supply entering Lake Mills by 15.6%. Uncertainty in these estimates was assumed to be 50%.

Geomorphic change in the river channel and floodplain were measured using the techniques of East *et al*.^22^, supplemented with DEMs generated from lidar and photogrammetry data from WY 2014–2016. East *et al*.^22^ used repeated total-station surveys and terrestrial lidar scans at fixed profile stations, longitudinal surveys of the thalweg elevation downstream of Lake Mills using RTK-GPS and acoustic sounders, physical sampling and analyses of sediment deposited in and along the channel and within the floodplain^69^, water-surface elevation monitoring with pressure and radar transducers along the channel margins at 28 stations, aerial lidar-based DEMs, and SfM-derived orthoimagery and digital elevation models.

These data were integrated into reach-based calculations of volumetric change both within and immediately outside of the active channel (combined and termed “Mainstem” for the purpose of Fig. 3) and in the broader floodplain using several different computational methods to incorporate a range of potential sedimentation patterns^22^. These methods agreed within 25% and for this paper, we used East *et al*.^22^ for 2012–2013 and method M2 of East *et al*.^22^ for estimates of active channel change for 2014–2016 values. For 2013–2016, floodplain and river sediment volumes were calculated from merged DEMs derived from lidar and photogrammetry. To calculate floodplain sediment storage, lidar data flown during seasons with minimal leaf extent were used to create DEMs of difference, and the analysis area was limited to floodplain inundated during the subject water year. For WY 2014 the floodplain was modeled using data from a 7 November 2014 lidar flight because higher flows were reached in WY 2013 than by that point in WY 2014, and no other floodplain elevation data were available. Uncertainty was estimated for elevation change in floodplain areas using multiple control sites which were known not to have been inundated.

To increase DEM accuracy in river reaches with low ground control point density, systematic error was quantified and subtracted using methods after Brasington *et al*.^70^. An error model was developed for photogrammetry DEMs using a dense series of bare earth control points in common with lidar flights, spaced at 50–100 m intervals along the river corridor. The difference between the photogrammetry and lidar surface models was calculated for these points and an error surface was generated using an empirical Bayesian kriging algorithm with a cell size of 20 m and a linear semivariogram composed of local models with ≤75 points, a local model overlap factor of 2, and 100 simulated semivariograms per model. The error model was subtracted from the photogrammetry DEM before merging with lidar data. Volume-change results were converted to sediment masses using a range of estimated bulk densities^22^. These results were summed into values representing the middle and lower reaches (Fig. 1), respectively.

Coastal change was measured from surveys of (1) nearshore bathymetry from personal watercraft equipped with single-beam sonar and differential GPS operating in real-time kinematic (RTK) mode, and (2) beach topography from RTK-GPS equipment mounted on backpacks and manually hiked along the beach^35^. Surveys were conducted primarily along a series of cross-shore transects spaced ~30 m apart and extending from approximately −14 m water depth to the landward side of the beach berm, although secondary data were collected throughout the study area to better characterize geomorphic variability between the transects. The surveys focused on the primary region of coastal delta growth, which extended ~2 km west and ~3 km east of the river mouth. Additional survey data and boat-based interferometric sidescan sonar were collected along the entire littoral cell extending more than 5 km west and 12 km east of the river mouth^35,71^.

Coastal DEMs were derived from gridding the survey data, and volume changes computed by differencing these DEMs. Total uncertainty of the DEMs was calculated to be 0.13 m. Beach and nearshore sediment samples were collected around the coastal delta during each survey and analyzed for grain-size distributions. Grain-size-dependent bulk densities were applied to the volume-change results to calculate change in sediment mass^33^. Gross erosion and deposition between each survey was also calculated; elevation change in the former estuary was estimated from land-based topographic surveys and rod sedimentation elevation table (RSET) measurements^27,33,38^.

The 5-yr sediment balance was computed by summing all of these elements and accounting for uncertainty in the measurements^33^. A supplementary table providing both annual values and the 5-yr sediment balance is provided in the Supplementary information, and forms the basis for Fig. 3.

Froude number, *Fr*, values at five USGS gauges on the Olympic Peninsula were determined using the equation^72,73^ *Fr* = [(*Q*^2^*w)/g*A^3^)]^0.5^, where *Q* is discharge, *w* is wetted top width of the channel, *g* is gravity, and *A* is the wetted cross-sectional area of the channel. In addition to the Elwha River at McDonald Bridge (station 12045500) gauges included the Dungeness River near Sequim, WA (station 12048000), Hoh River at U.S. Highway 101 (station 12041200), Queets River near Clearwater, WA (station 12040500), and Quinault River at Lake Quinault (station 12039500). Dimensions of the wetted channel for discrete discharge measurements at these USGS gauges are available through the National Water Information System (https://waterdata.usgs.gov/nwis).

The changes in river stage through time at gauging station 12045500 (Elwha River at McDonald Bridge, Rkm 13.5) and a pressure transducer at Rkm 5.5 were determined from the shifts in their stage relative to stage recorded at the diversion weir gauge (USGS gauge 12046260) measured through time^22,74^. The diversion gauge was selected as a reference against which to compare other stage gauges because the structure was engineered to minimize the change in stage during sediment releases associated with dam removal.

To measure sinuosity and braiding, we digitized channel centerlines on the orthoimages using ArcGIS, while viewing the images at a scale of 1:500. Channel centerlines were digitized for all wetted channels with surface-water connection to the mainstem channel (i.e., the channel that appeared to be carrying the largest proportion of flow). Mainstem sinuosity was calculated as the length of the mainstem channel divided by the straight-line down-valley distance, considering the middle and lower Elwha River reaches separately (Fig. 1). Braiding index was calculated as the sum of all channel lengths divided by the mainstem length^75^, again considering the middle and lower reaches separately.

## Electronic supplementary material

Supplementary Table 1

## Data Availability

Data presented herein, including daily sediment loads, digital elevation models, dam elevations, bedload estimates, orthomosaics, streamgage measurements, suspended sediment concentration data, and calculations of upstream sediment contributions to Lake Mills, can be viewed and downloaded from the USGS ScienceBase repository at 10.5066/F7PG1QWC.
