# Supplementary material for: Morphodynamic evolution following sediment release from the world’s largest dam removal
Source: Sci Rep. 2018 Sep 5;8:13279. doi: 10.1038/s41598-018-30817-8 (PMC6125403; doi:10.1038/s41598-018-30817-8)
Supplement: Supplementary file 1 — Supplementary Table 1 [file 41598_2018_30817_MOESM1_ESM.pdf]

Morphodynamic evolution following sediment release from the world's largest dam removal

AUTHORS

Andrew C. Ritchie[1]*, Jonathan A. Warrick[1], Amy E. East[1], Christopher S. Magirl[2], Andrew W. Stevens[1], Jennifer A Bountry[3], Timothy J Randle[3], Christopher A Curran[4], Robert C Hilldale[3], Jeffrey J. Duda[5], Ian M. Miller[6], George R. Pess[7], Melissa M. Foley[1], Randall McCoy[8] & Andrea S. Ogston[9]

**Corresponding Author*

AFFILIATIONS

1. Pacific Coastal and Marine Science Center, United States Geological Survey, Santa Cruz, CA, USA
2. Arizona Water Science Center, United States Geological Survey, Tucson, AZ, USA
3. Sedimentation and River Hydraulics Group, Technical Service Center, United States Bureau of Reclamation, Denver, CO, USA
4. Washington Water Science Center, United States Geological Survey, Tacoma, WA, USA
5. Western Fisheries Research Center, United States Geological Survey, Seattle, WA, USA
6. Washington Sea Grant, Olympic Peninsula Field Office, Port Angeles, WA, USA
7. Northwest Fisheries Science Center, National Marine Fisheries Service, National Oceanic and Atmospheric Administration, Department of Commerce, Seattle, WA, USA
8. Natural Resources Department, Lower Elwha Klallam Tribe, Port Angeles, WA, USA
9. School of Oceanography, University of Washington, Seattle, WA, USA

Supplemental Materials Table 1. Sediment mass balance for the Elwha River during the first five years following dam removal; all values have been rounded to two significant figures

			**Sediment Mass (kt)**					
**Location (listed in upstream to downstream order)**	**Type**	**2σ (%)**	**Year 1 (WY12)**	**Year 2 (WY13)**	**Year 3 (WY14)**	**Year 4 (WY15)**	**Year 5 (WY16)**	**5-yr Total** *(uncertainty, kt)*
1. Flux from upper watershed	**Source**	**75%***	92	110	100	370	490	**1200** *(300-2100)*
2. Net change in Lake Mills	**Source**	**20%**	190	8800	3500	2400	1200	**16,100** *(12,900-19,300)*
3*(a)*. Middle reach deposition, mainstem channel	**Sink**	**19%**	0	290	280	160	26	**380** *(310-450)*
3*(b)*. Middle reach deposition, floodplain channels	**Sink**	**57%**	0	160	14	6.5	56	**240** *(100-380)*
4. Tributary sediment inputs	**Source**	**75%***	14	17	16	58	76	**180** *(46-320)*
5. Net change in Lake Aldwell	**Source**	**18%**	1100	220	300	1100	450	**3200** *(2600-3800)*
6*(a)*. Suspended-sediment discharge at diversion weir	**Flux**	**49%**	930	5400	3000	3000	2000	**14,300** *(7300-21,000)*
6*(b)*. Bedload sediment discharge at diversion weir	**Flux**	**91%**	160	1700	990	1000	680	**4600** *(410-8700)*
7*(a)*. Lower reach deposition, mainstem channel	**Sink**	**17%**	62	560	150	(88)	300	**980** *(820-1200)*
7*(b)*. Lower reach deposition, floodplain channels	**Sink**	**58%**	31	103	98	150	160	**540** *(230-850)*
8. Estuary deposition	**Sink**	**100%***	11	5	1.3	11	(5.4)	**22** *(0-60)*
9. Coastal deposition	**Sink**	**29%**	170	3200	600	900	570	**5400** *(3900-7000)*
10. Marine Dispersal**	**Sink**	**r.s.e.****	1200	4900	2800	3100	1100	**13,100** *(9400-17,000)*

*Uncertainty values were not measured, so values are assumed.
**Marine dispersal values computed by sediment balance summation, uncertainty computed by the root sum of the squared errors (r.s.e.).
